# Intention and practice on breastfeeding among pregnant mothers in Malaysia and factors associated with practice of exclusive breastfeeding: A cohort study

**DOI:** 10.1371/journal.pone.0262401

**Published:** 2022-01-07

**Authors:** Nurul Mursyidah Shohaimi, Majidah Mazelan, Kanesh Ramanathan, Mai Shahira Meor Hazizi, Yan Ning Leong, Xiang Bin Cheong, Subashini Ambigapathy, Ai Theng Cheong

**Affiliations:** 1 Buntong Health Clinic, Ministry of Health Malaysia, Ipoh, Perak, Malaysia; 2 Jelapang Health Clinic, Ministry of Health Malaysia, Ipoh, Perak, Malaysia; 3 Tanjung Malim Health Clinic, Ministry of Health Malaysia, Tanjung Malim, Perak, Malaysia; 4 Pantai Remis Health Clinic, Ministry of Health Malaysia, Pantai Remis, Perak, Malaysia; 5 Simee Health Clinic, Ministry of Health Malaysia, Ipoh, Perak, Malaysia; 6 Chemor Health Clinic, Ministry of Health Malaysia, Ipoh, Perak, Malaysia; 7 Department of Family Medicine, Faculty of Medicine and Health Sciences, Universiti Putra Malaysia, Serdang, Selangor, Malaysia; UCSI University, MALAYSIA

## Abstract

**Background:**

Exclusive breastfeeding rate in Malaysia is low despite its known health benefits. This study aims to determine the prevalence of intention to breastfeed among pregnant mothers, the prevalence of exclusive breastfeeding practice after delivery, and factors associated with exclusive breastfeeding practice.

**Methods:**

This was a prospective cohort study. All pregnant women at 36 weeks gestation or above from 17 antenatal health clinics in an urban district were invited to participate in the study. A self-administered questionnaire was used, encompassing sociodemographic, breastfeeding knowledge, attitude, and intention towards the practice of breastfeeding. The participants were followed up one month post-natal for their practice of breastfeeding via telephone or during their post-natal follow-up appointment.

**Results:**

483 pregnant mothers participated in the study initially. 462 (95.7%) were contactable after one month. 99.4% (459/462) of participants intended to breastfeed. 65.4% (302/462) of participants practiced exclusive breastfeeding. There was no significant association between intention and practice of exclusive breastfeeding. Multiple logistic regression analysis shows, pregnant mothers with high breastfeeding knowledge (AOR = 1.138; 95% CI 1.008–1.284) and Malay ethnicity (AOR = 2.031; 95% CI 1.066–3.868) were more likely to breastfeed their infant exclusively.

**Conclusions:**

Prevalence of exclusive breastfeeding practice at one month in the studied district was 65.4%. Malay mothers and mothers with high breastfeeding knowledge were more likely to breastfeed exclusively. Thus, we recommend targeted intervention towards non-Malay mothers and increasing breastfeeding knowledge to all pregnant mothers.

## Introduction

Breast milk is the best gift from a mother to a baby. Breastfeeding has long been associated with a reduced risk of diseases in infants and mothers [1]. Breast milk is the healthiest infant nutrition, especially during the first six months after birth [2]. Breast milk helps build a child’s and the new mother’s immunity, thus leading to increased prevention of illnesses [1]. Research has also informed that formula drinks are often associated with the rise in childhood infections such as diarrhea, ear infections, type 2 diabetes, and childhood obesity [3]. Women who breastfed were noted to have a significantly lesser chance of developing breast or ovarian cancer as than who never breastfed [4]. The practice of breastfeeding increases the sense of closeness between a mother and child, thus making their attachment stronger during the crucial developmental years [5]. Studies also found a positive correlation between the increased practice of breastfeeding and lower risk for postpartum depression [6]. In addition to that, breastfeeding also has an economic advantage as it is inexpensive, and by decreasing the risk of illnesses of an infant and mother, breastfeeding helps to reduce their medical expenses [7].

Exclusive breastfeeding is defined as an infant receiving only breast milk without additional food or drink, not even water [8]. The World Health Organization (WHO) recommends exclusive breastfeeding for the first six months of life and continued breastfeeding until the age of 2 years old with complimentary food [9].

Malaysia is a middle-income country in Southeast Asia. Malaysia acknowledges the recommendation of breastfeeding and revised its existing National Breastfeeding Policy in 2006 [10]. Multiple strategies are being taken to promote exclusive breastfeeding practices such as Baby-Friendly Hospital Initiative, Baby-Friendly Clinic Initiative, training of health staff, 90-day maternity leave in the governmental sector and implementation, of the Code of Ethics for Marketing of Infant Foods and Related Products. Our national Key Performance Index set for exclusive breastfeeding for up to 6 months is 58%. However, based on the latest National Health Morbidity Survey in 2016, the achievement was 47.1%. Overall, the prevalence of continued breastfeeding among children aged 20–23 months old is even lower at 39.4% [10].

Many local studies showed that only one-third of mothers breastfeed their child exclusively for the first six months [11, 12]. A study in seven private hospitals in Malaysia showed that 40% of Malaysian mothers chose to exclusively breastfeed, although they were well aware of the practice of breastfeeding [11]. Another study conducted in two government primary healthcare clinics, including their subsidiary community clinics in Klang district had reported the prevalence of exclusive breastfeeding on infants aged between 1 and 6 months was 43.1%, with factors positively influencing breastfeeding among mothers being: living in a rural area, Malay ethnicity, non-working mothers, non-smoker, multiparity, term infants, bed-sharing between mother and infant, and supportive husbands [12].

Little study has been done to determine the intention of breastfeeding made during pregnancy. A local study done in an urban government hospital revealed that most pregnant Malaysian mothers only intended to practice exclusive breastfeeding to their infants for around 14 weeks after delivery [13]. We were unable to find local literature investigating the intention of pregnant mothers to practice breastfeeding in primary care settings. In this study, we aimed to determine the association between the intention of practice breastfeeding among pregnant mothers and their actual practice of breastfeeding after delivery and evaluate the factors associated with exclusive breastfeeding.

## Materials and methods

### Study design and data collection

This was a prospective cohort study. All pregnant mothers at 36 weeks gestation or above who attended the 17 antenatal clinics (S1 List) at government primary healthcare clinics in Kinta District from 14^th^ August 2019 to 16^th^ October 2019 were invited to participate in the study.

Kinta is the biggest and populated district in the state of Perak, and public primary healthcare clinics are the main providers for antenatal and postnatal care. Out of 17 clinics included in the study, only one clinic does not fulfill baby-friendly clinic criteria as it is under renovation therefore unable to accommodate a breastfeeding room or corner to encourage breastfeeding. Besides providing facilities, to become a baby-friendly clinic all healthcare personnel in the clinic must be trained with skills to encourage, guide, and assist mothers on exclusive breastfeeding [14].

The inclusion criteria were to be able to read and understand either English language or Bahasa Malaysia (Malaysia’s national language) and aged 18 years old and above. Pregnant mothers who were non-citizens, with retroviral disease, or undergoing chemotherapy or radiation treatment were excluded from the study.

Sample sizes were calculated using Sampsize, based on the prevalence of a local study whereby the prevalence of exclusive breastfeeding at one month was 63.3% [12]. With a 95% confidence level and a 5% margin of error, the minimum sample size required was 446 after taking into account a non-respondent rate of 20%.

All eligible pregnant mothers were identified at the registration counter of the respective clinic by a health care assistant or community nurse who was not involved with the antenatal care of pregnant mothers. Researchers had trained the data collectors to familiarize themselves with the research instruments and processes of recruitment. The participant information sheet was explained to these pregnant mothers, and written consent was obtained from each participant. Each participant was required to complete a self-administered questionnaire, and they were given a choice either to complete the Malay or English questionnaire. Participants were followed up one month post-delivery either via phone call or during the post-natal follow-up appointment to inquire about their infants’ feeding status.

Two instruments were used for data collection. The first instrument was a self-administered questionnaire given to eligible and consented participants. The self-administered questionnaires were divided into four sections (S1 File). Section A of the questionnaire encompassed the sociodemographic data of the participants, such as age, ethnicity, marital status, educational status, occupational status, and parity. Section B of the questionnaire consisted of 14 questions regarding knowledge on breastfeeding and section C consisted of 8 questions on the attitude towards breastfeeding. Both sections B and C were adapted from a locally published article [15]. Section D assessed the intention of participants to breastfeed their babies with a dichotomous (yes or no) question.

The second instrument was a questionnaire that was asked to participants who were contacted at one month post-delivery. Researchers recorded the responses to assess the practice of breastfeeding using a dichotomous question that inquired whether they breastfeed their infant or not and if yes, whether they exclusively breastfeed or practiced mixed breast and formula feeding. In this study, exclusive breastfeeding was defined as infants receiving only breast milk from their mother (either directly from the breast or expressed) without other liquids or solids except drops or syrups consisting of vitamins, mineral supplements, or medicines over one month.

The content validation of the questionnaire was assessed by an expert panel which consisted of Family Medicine specialists who practice in academic and government health clinic settings. The questionnaire was translated from English to Malay via forward and backward translation by school teachers proficient in both languages. A pilot study was carried out with a total of 30 pregnant mothers to check the understanding and reliability of the questionnaire and examine practical issues in recruiting participants. The pilot study revealed that the Cronbach’s alpha of section B (breastfeeding knowledge) and section C (attitude) were 0.75 and 0.66 respectively, suggesting good and moderate reliability of the questionnaire.

### Ethical issues

Ethical approval of the study was obtained from Medical Research and Ethics Committee (NMRR-19-915-47684). The participant information sheet was explained to these pregnant mothers, and written consent was obtained from each participant.

### Data analysis

The dependent variable was the practice of exclusive breastfeeding. The independent variables were age, ethnicity, education level, work, marital status, parity, knowledge, attitude, and intention towards breastfeeding.

For questions on breastfeeding knowledge and attitude, answers were scored with correct or positive answers given a 1 mark without any negative marking involved. The total score of breastfeeding knowledge ranged from 0 to 14 with higher scores indicating better knowledge about breastfeeding. The total score of attitude ranged from 0 to 8. Higher scores indicated a more positive attitude towards exclusive breastfeeding.

Completed data were analyzed using SPSS Statistics version 25.0. Categorical data were described in frequency (n) and percentage (%). The continuous data (age, breastfeeding knowledge and attitude scores) were not normally distributed based on the Shapiro-Wilks normality test; hence data were presented in the median and interquartile range (IQR).

To assess the association between the practice of exclusive breastfeeding with intention, breastfeeding knowledge, attitude, and sociodemographic factors, the Mann-Whitney test and chi-squared test were used in bivariate analyses. The level of statistical significance was set as p<0.05. Variables that were found significant in the bivariate analyses (ethnicity, breastfeeding knowledge, and attitude score) and found to be significant in literature were retained (age, ethnicity, education level, work status, breastfeeding knowledge, and attitude) and further analyzed using multiple logistic regression to calculate respective odds ratios and associated 95% confidence intervals for predictors of exclusive breastfeeding practice.

## Results

A total of 483 pregnant mothers were initially enrolled in this study. However, 21 participants were unable to be contacted one month post-delivery. The response rate for the follow-up was 95.65% (462/483). Analyses were conducted on 462 responses from the one-month post-natal appointment. Table 1 shows the sociodemographic information of all the respondents. The respondents were predominantly 20–34 years old (84.4%) with a median age of 30 (IQR 6). The majority of the participants were Malay (62.6%), married (99.1%), received tertiary education (54.8%), unemployed (39.8%), and multigravida (60.8%).

**Table 1:** Sociodemographic characteristics of participants (N = 462).

Variable	n	%	Median(IQR)
**Age**			30(6)
0–19	2	0.4	
20–34	390	84.4	
35–44	70	15.2	
**Ethnicity**			
Malay	289	62.5	
Chinese	108	23.4	
Indian	49	10.6	
Others	16	3.5	
**Marital status**			
Married	458	99.1	
Single	4	0.9	
**Education**			
Primary	19	4.1	
Secondary	190	41.1	
Tertiary	253	54.8	
**Work**			
Government	94	20.4	
Private	146	31.6	
Self	38	8.2	
Unemployed	184	39.8	
**Parity**			
Primigravida	181	39.2	
Multigravida	281	60.8	

IQR = Interquartile Range.

The median score for breastfeeding knowledge was 13 (IQR = 3.0), and the median score for attitude was 7 (IQR = 1.0). 99.4% (459/462) of pregnant mothers had the intention to breastfeed their babies postpartum. At one month post-delivery, 65.4% (302/462) of them practiced exclusive breastfeeding, while 35.6% did not practice exclusive breastfeeding.

Table 2 shows the Mann-Whitney test results of exclusive breastfeeding practice by age, breastfeeding knowledge, and attitude. There were significant differences in breastfeeding knowledge (p <0.001) and attitude (p = 0.020) among mothers who practiced exclusive breastfeeding compared to the mothers who did not exclusively breastfeed. Those mothers who exclusively breastfed had higher breastfeeding knowledge and attitude scores.

**Table 2:** Practice of exclusive breastfeeding by age, breastfeeding knowledge score, and attitude score (N = 462).

Variables	Breastfeeding Practice	Median (IQR)	Mean Rank	Z	p-value
**Age**	Exclusive	29.5 (6.00)	230.57	-0.206	0.837
	Not Exclusive	30.0 (7.00)	233.25		
**Breastfeeding knowledge Score**	Exclusive	13.0 (2.00)	251.00	-4.418	<0.001*
	Not Exclusive	12.0 (2.75)	194.70		
**Attitude Score**	Exclusive	7.0 (1.00)	241.31	-2.324	0.020 *
	Not Exclusive	7.0 (2.00)	212.99		

There is a significant association if *p <0.05.

IQR = Interquartile Range.

Z = Mann-Whitney statistic value.

Table 3 shows the chi-squared test results to assess the association between intention and sociodemographic characteristics of participants with the practice of exclusive breastfeeding. 2 (66.7%) respondents out of the 3 who did not intend to breastfeed, practiced exclusive breastfeeding. The analysis showed no significant association between intention and practice of exclusive breastfeeding (p = 1.000). Ethnicity shows a significant association with the practice of exclusive breastfeeding (p = 0.025). Most Malay respondents (69.9%) practiced exclusive breastfeeding, while 51.0% of Indian respondents did not practice exclusive breastfeeding.

**Table 3:** Association of intention and sociodemographic characteristics of participants with the practice of exclusive breastfeeding (N = 462).

Variable	Exclusive		Not Exclusive			χ^2^	p-value
	n	%	n		%		
**Ethnicity**						9.354	0.025 *
Malay	202	69.9		87	30.1		
Chinese	66	61.1		42	38.9		
Indian	24	49.0		25	51.0		
Others	10	62.5		6	37.5		
**Marital status**						2.904	0.122 ^‡^
Married	301	99.7		157	0.3		
Single	1	98.1		3	1.9		
**Education**						0.273	0.872
Primary	12	63.2		7	36.8		
Secondary	122	64.2		68	35.8		
Tertiary	168	66.4		85	33.6		
**Work**						0.178	0.981
Government	60	63.8		34	36.2		
Private	95	65.1		51	34.9		
Self	25	65.8		13	34.2		
Unemployed	122	66.3		62	33.7		
**Parity**						2.148	0.143
Primigravida	111	61.3		70	38.7		
Multigravida	191	68.0		90	32.0		
**Intention**						0.002	1.000^‡^
Yes	300	65.4		159	34.6		
No	2	66.7		1	33.3		

There is a significant association if *p< 0.05.

χ^2^ = Chi square.

^‡^ Fisher’s Exact Test.

Table 4 shows the results of multiple factors which were explored using multiple logistic regression analysis. It is found that mothers with higher breastfeeding knowledge scores (AOR  =  1.138; 95% CI 1.008–1.284) were significantly more likely to breastfeed their children exclusively. The odds for mothers of Malay ethnicity to exclusively breastfeed was two times more than mothers of Indian ethnicity (AOR = 2.031; 95% CI 1.066–3.868).

**Table 4:** Multiple logistic regression of sociodemographic, breastfeeding knowledge score, and attitude score in influencing exclusive breastfeeding (N = 462).

Variable	Odds ratio	95% CI		p-value
		Lower	Upper	
**Age**	0.989	0.945	1.036	0.648
**Ethnicity**				
Indian	ref			
Others	1.678	0.506	5.571	0.398
Chinese	1.568	0.778	3.162	0.208
Malay	2.031	1.066	3.868	0.031*
**Work**				
Unemployed	ref			
Government	0.706	0.395	1.262	0.240
Private	0.999	0.616	1.620	0.996
Self	1.204	0.557	2.605	0.637
**Education**				
Primary education	ref			
Secondary	1.247	0.442	3.521	0.677
Tertiary	1.343	0.470	3.837	0.582
**Breastfeeding knowledge Score**	1.138	1.008	1.284	0.036 *
**Attitude Score**	1.140	0.951	1.365	0.156

There is a significant association if *p < 0.05; Pseudo R square = 0.067.

CI = Confidence Interval.

ref = reference group.

## Discussion

We found that the practice of exclusive breastfeeding at one month postpartum in the Kinta district was 65.4% and not significantly associated with the intention to breastfeed. Significant associated factors of exclusive breastfeeding practice were Malay ethnicity (AOR = 2.031; 95% CI 1.066–3.868) and higher breastfeeding knowledge score (AOR  =  1.138; 95% CI 1.008–1.284).

In this study, the prevalence of exclusive breastfeeding for at least one month postpartum was 65.4%. This figure is higher compared to a local study conducted in government primary care clinics in Klang; the prevalence of exclusive breastfeeding at the first month postpartum was 63.3%, and the prevalence at the sixth month postpartum was 32.4% [12]. The Malaysia National Health Morbidity Survey in 2016 reported that the prevalence of exclusive breastfeeding at two months postpartum was 52.9% and 47.4% at fourth months postpartum [10]. We expected that the prevalence of exclusive breastfeeding would drop further if the study continued for six months due to mothers returning to work or lack of support from husbands [12].

The intention to breastfeed children was high (99.4%) in this study. The high number of pregnant mothers who intended to breastfeed may be attributed to the increased breastfeeding awareness programs at the health clinics or could be due to social desirability bias whereby the respondents could feel obliged to answer that they have the intention to breastfeed their child [16]. It was hypothesized that those with an intention to breastfeed during pregnancy would have practiced exclusive breastfeeding after delivery. However, this study showed no significant association between intention to breastfeed and the practice of exclusive breastfeeding. This was in contrast to a study done in the literature which found breastfeeding intention was a significant predictor of positive breastfeeding outcomes [17]. The discrepancy of the intention and actual breastfeeding practice could be due to overreporting of the intention, or the possibility of mothers facing challenges that hinder their exclusive breastfeeding. Literature had shown that barriers for mothers to breastfeed after delivery included perceived low milk quantity, separation of mother and infant, fatigue due to breastfeeding, and return to work [18].

Ethnicity and breastfeeding knowledge showed significant associations with the practice of exclusive breastfeeding in the bivariate analysis and remained significant in multiple logistic regression analysis after adjustment with other variables. Malay mothers were two times more likely to practice exclusive breastfeeding compared to Indian mothers. This finding was consistent with a local study conducted at rural health clinics where Malay mothers were found to be four times more likely than their non-Malay counterparts to practice exclusive breastfeeding [19].

Confinement is defined as restrictions on practices or diet after delivery, and the three major ethnic groups in Malaysia share similarities in their confinement practice, such as postpartum diet, massage and herbal baths [20]. The high likelihood for Malay mothers to practice exclusive breastfeeding could be due to the Malay post-partum confinement culture and good support from traditional massage practitioners who provide massage services and breastfeeding encouragement to the mothers during the confinement period [21]. A study in Singapore shows a higher prevalence of massage used by Malay mothers during the confinement period compared to Chinese and Indian mothers [22]. For the Chinese mothers, it is believed that mothers need to have enough rest during one month postpartum confinement period where Chinese mothers often do not room-in with their infants, contributing to the lower exclusive breastfeeding rates [22–24]. Our study found that about half of Indian mothers (51%) did not exclusively breastfeed their infants at one month. Previous literature reported that women of the Indian ethnic group were four times more likely to not breastfeed exclusively at four weeks than Malay women [25]. Hence exclusive breastfeeding counseling and promotion during the antenatal period should focus more on non-Malay mothers, especially mothers of Indian ethnicity.

We found that higher breastfeeding knowledge was associated with a higher likelihood of practicing exclusive breastfeeding. This result was consistent with findings from the literature. Studies in Ghana and the United Arab Emirates (OR 5.9; 95% CI 2.6–13.3), (AOR 1.25; 95% CI 1.04–1.50) respectively have reported that those with higher breastfeeding knowledge were associated with the likelihood of exclusive breastfeeding [26, 27]. A local study in two Kelantan hospitals also found that breastfeeding knowledge is a significantly associated factor for exclusive breastfeeding (AOR 1.06; 95% CI 1.01–1.11) [28]. Therefore, educating pregnant mothers on the benefits and importance of exclusive breastfeeding may help to increase the practice of exclusive breastfeeding, and this could be emphasized during antenatal education to all pregnant women.

### Strengths and limitations

The strength of the study is the coverage of all 17 government primary healthcare clinics in the Kinta district. We used a prospective cohort study design to examine the actual practice of breastfeeding post-delivery to avoid recall bias. Limitations of this study include the self-administered questionnaire, which was subject to recall and social desirability bias. The questionnaire adopted for assessment of breastfeeding knowledge and attitude in our study lacked construct validity; nevertheless, we had undergone the process of content validity and reliability. This study was conducted only up to one month postpartum due to resource constraints. If the study was conducted up to six months postpartum, the prevalence of exclusive breastfeeding practice that fulfills the recommended duration of six months could be determined. This study did not include other factors that could influence exclusive breastfeeding duration and reasons for exclusive breastfeeding discontinuation such as husband attitude and workplace support. Another limitation of this study includes a relatively small sample size of non-Malay (specifically Indian) participants. Thus, the generalizability of this study to the general population needs to be interpreted with caution. However, the ethnic proportion in this study is comparable to Malaysia’s overall ethnic composition [29].

## Conclusion

The prevalence of exclusive breastfeeding practices at one month in the Kinta district was 65.4%, and Malay mothers and mothers with higher breastfeeding knowledge were more likely to breastfeed exclusively. Thus, we recommend targeted intervention towards non-Malay mothers and increasing breastfeeding knowledge to all pregnant mothers.

## Supporting information

S1 List List of participating clinics. (DOCX)

S1 File Questionnaire for data collection. (DOCX)
