# Supplementary material for: Intention and practice on breastfeeding among pregnant mothers in Malaysia and factors associated with practice of exclusive breastfeeding: A cohort study
Source: PLoS One. 2022 Jan 7;17(1):e0262401. doi: 10.1371/journal.pone.0262401 (PMC8741045; doi:10.1371/journal.pone.0262401)
Supplement: S1 List — (DOCX) [file pone.0262401.s001.docx]

S1 List: List of participating clinics.

1. Greentown Health Clinic
2. Manjoi Health Clinic
3. Jelapang Health Clinic
4. Tanjung Rambutan Health Clinic
5. Chemor Health Clinic
6. Gunung Rapat Health Clinic
7. Buntong Health Clinic
8. Menglembu Health Clinic
9. Pasir Pinji Health Clinic
10. Simee Health Clinic
11. Tronoh Health Clinic
12. Tanjung Tualang Health Clinic
13. Batu Gajah Maternal & Child Health Clinic
14. Simpang Pulai Maternal & Child Health Clinic
15. Pengkalan Pegoh Maternal & Child Health Clinic
16. Pusing Maternal & Child Health Clinic
17. PGA Kinta Maternal & Child Health Clinic
