# Supplementary material for: Intention and practice on breastfeeding among pregnant mothers in Malaysia and factors associated with practice of exclusive breastfeeding: A cohort study
Source: PLoS One. 2022 Jan 7;17(1):e0262401. doi: 10.1371/journal.pone.0262401 (PMC8741045; doi:10.1371/journal.pone.0262401)
Supplement: S1 File — (DOCX) [file pone.0262401.s002.docx]

S1 File: The ‘Intention and practice on breastfeeding among pregnant mothers and factors associated with exclusive breastfeeding’ questionnaire.

**The information from this study will be used strictly for research purposes only and your identity will not be disclosed.**

SERIAL NUMBER: _____________ Date*:* _____________

Please tick (√) where appropriate and answer all the questions.

PART A: SOCIODEMOGRAPHIC DATA

1. Age: _____
2. Ethnicity

Malay

Chinese

Indian

Others

1. Marital status

Married

Unmarried

1. Educational status

None

Primary

Secondary

Tertiary

1. Occupational status

Government employee

Private employee

Daily labourer

Self-employed

Housewife

Others

1. Parity and gravidity.

Primigravida (1st pregnancy)

Multigravida

(2nd pregnancy or above)

PART B: KNOWLEDGE OF BREASTFEEDING

1. What is the best food for the infant? Tick only, one answer?

Breast milk

Formula milk

Others

I don’t know

1. Is breast milk good for infant's resistance towards diseases?

Yes

No

I don’t know

1. Does breastfeeding increase maternal and child bonding?

Yes

No

I don’t know

1. Is breastfeeding convenient and economical?

Yes

No

I don’t know

1. Does breastfeeding help mothers to recover from childbirth?

Yes

No

I don’t know

1. Is breast milk good to avoid infant allergies?

Yes

No

I don’t know

1. Is breast milk a well-balanced nourishing food?

Yes

No

I don’t know

1. Does breast milk help with infant teeth development?

Yes

No

I don’t know

1. Does breast milk fill up the stomach more easily?

Yes

No

I don’t know

1. Does breastfeeding help mothers lose weight?

Yes

No

I don’t know

1. Does breastfeeding need to be stopped when the baby and/or mother is sick?

Yes

No

I don’t know

1. Should clear fluid be given to babies who are exclusively breastfeeding?

Yes

No

I don’t know

1. Is colostrum good?

Yes

No

I don’t know

1. What is the age of weaning from breastfeeding?

2 months

6 months

8 months

PART C: ATTITUDE OF BREASTFEEDING

1. Is breastfeeding easier than feeding infants formula?

Yes

No

I don’t know

1. Does breastfeeding have a negative effect on marital relationships?

Yes

No

I don’t know

1. Do breastfeeding mother have difficulty taking care of their families?

Yes

No

I don’t know

1. Can you breastfeed babies with modesty anywhere?

Yes

No

I don’t know

1. Can you start breastfeeding straight after delivery?

Yes

No

I don’t know

1. Is there a ban in the use of bottles and teats in any hospital?

Yes

No

I don’t know

1. Does your community encourage breastfeeding?

Yes

No

I don’t know

1. Should you stop breastfeeding if your husband discourages?

Yes

No

I don’t know

PART D: INTENTION OF BREASTFEEDING

1) Do you intend to breastfeed your child after delivery?

Yes

No

We are sincerely grateful for your willingness to participate in this study. We appreciate all your opinions and knowledge. In case you have any questions about this questionnaire, please contact:

Dr.Kanesh A/L Ramanathan

E-mail: **kanesh1060@gmail.com**

**ONE MONTH POSTNATAL QUESTIONNAIRE**

SERIAL NUMBER: _____________ Date*:* _____________

NAME: _____________ CONTACT NUMBER: _____________

1) Are you currently breastfeeding your child?

Yes

No

1. If yes,

Exclusive

Mixed
